# Effects of hospital funding reform on wait times for hip fracture surgery: a population-based interrupted time-series analysis

**DOI:** 10.1186/s12913-021-06601-2

**Published:** 2021-06-13

**Authors:** Daniel Pincus, Jessica Widdifield, Karen S. Palmer, J. Michael Paterson, Alvin Li, Anjie Huang, David Wasserstein, Lauren Lapointe-Shaw, Adalsteinn Brown, Monica Taljaard, Noah M. Ivers

**Affiliations:** 1 Department of Surgery, University of Toronto, 149 College Street, Room 508-A, ON M5T 1P5 Toronto, Canada; 2 ICES, Toronto, Canada; 3 Institute of Health Policy, Management and Evaluation, University of Toronto, Toronto, Canada; 4 Holland Bone & Joint Program, Sunnybrook Research Institute, Toronto, Canada; 5 Women’s College Research Institute, Women’s College Hospital, Toronto, ON Canada; 6 Faculty of Health Sciences, Simon Fraser University, Burnaby, BC Canada; 7 Ottawa Hospital Research Institute, Ottawa, Ontario Canada; 8 Department of Medicine, University of Toronto, Toronto, Ontario Canada

**Keywords:** Epidemiology, Health Policy, Femur, Fracture Fixation, Internal, Retrospective studies, Ontario, Canada, Orthopaedic Surgery

## Abstract

**Background:**

Health care funding reforms are being used worldwide to improve system performance but may invoke unintended consequences. We assessed the effects of introducing a targeted hospital funding model, based on fixed price and volume, for hip fractures. We hypothesized the policy change was associated with reduction in wait times for hip fracture surgery, increase in wait times for non-hip fracture surgery, and increase in the incidence of after-hours hip fracture surgery.

**Methods:**

This was a population-based, interrupted time series analysis of 49,097 surgeries for hip fractures, 10,474 for ankle fractures, 1,594 for tibial plateau fractures, and 40,898 for appendectomy at all hospitals in Ontario, Canada between April 2012 and March 2017. We used segmented regression analysis of interrupted monthly time series data to evaluate the impact of funding reform enacted April 1, 2014 on wait time for hip fracture repair (from hospital presentation to surgery) and after-hours provision of surgery (occurring between 1700 and 0700 h). To assess potential adverse consequences of the reform, we also evaluated two control procedures, ankle and tibial plateau fracture surgery. Appendectomy served as a non-orthopedic tracer for assessment of secular trends.

**Results:**

The difference (95 % confidence interval) between the actual mean wait time and the predicted rate had the policy change not occurred was − 0.46 h (-3.94 h, 3.03 h) for hip fractures, 1.46 h (-3.58 h, 6.50 h) for ankle fractures, -3.22 h (-39.39 h, 32.95 h) for tibial plateau fractures, and 0.33 h (-0.57 h, 1.24 h) for appendectomy (Figure 1; Table 3). The difference (95 % confidence interval) between the actual and predicted percentage of surgeries performed after-hours − 0.90 % (-3.91 %, 2.11 %) for hip fractures, -3.54 % (-11.25 %, 4.16 %) for ankle fractures, 7.09 % (-7.97 %, 22.14 %) for tibial plateau fractures, and 1.07 % (-2.45 %, 4.59 %) for appendectomy.

**Conclusions:**

We found no significant effects of a targeted hospital funding model based on fixed price and volume on wait times or the provision of after-hours surgery. Other approaches for improving hip fracture wait times may be worth pursuing instead of funding reform.

**Supplementary Information:**

The online version contains supplementary material available at 10.1186/s12913-021-06601-2.

## Background

Health care funding reforms are occurring worldwide in an attempt to improve patient outcomes and decrease medical costs [1, 2]. Among the 37 OECD member countries, for example, total health expenditures as a percent of GDP ranged in 2019 from 4.4 % in Turkey to 17.0 % in the United States, with Canada at 10.8 % [3]. Similarly, evidence shows that the everyday practice of medicine is “characterised by wide variations that have no basis in clinical science”, leading to variability in patient outcomes [4].

These reforms include “Quality-Based Procedures (QBPs)”, a hospital funding initiative implemented in Ontario, Canada. QBPs, a novel variant of activity-based funding (ABF) [5], are a procedure- and diagnosis-specific approach to funding hospitals. They involve a pre-set price per episode of care, coupled with a best practice clinical pathway for each of the pre-specified diagnoses and procedures. QBPs replaced part of each hospital’s global budget—the annual operating revenue—with a prospectively determined amount targeted at those procedures and diagnoses [6, 7]. The funding for QBPs was carved out of each hospital’s global budget, and then reallocated back as a fixed price for a fixed volume of targeted care.

Hip fracture surgery was among the procedures selected for targeted funding through the QBP mechanism because of its relatively high mortality rate, [8] cost to the health care system, [9, 10] and variation in care associated with these injuries [11]. In parallel with the QBP funding change, handbooks outlining best practice clinical pathways for each QBP, including hip fracture surgery, were developed and disseminated to encourage standardization of care. [12] The clinical pathway was intended to encourage reduced wait times for surgery [1, 2] because delays for hip fracture surgery are associated with increased complications, [13–15] medical costs, [10] and length of stay [10]. The theory was that because longer waits for hip fracture repair increases complications and costs, targeted QBPs funding would encourage reduced waiting times. Prior research has examined this program theory and the rationale underpinning QBPs [1, 2].

To date, whether targeted hospital funding reform has had its intended effect of reducing hip fracture surgery wait times has not been assessed. The aim of this study was to determine whether implementation of the QBP funding reform, along with dissemination of best practice clinical pathways, in a large population-based cohort from Ontario, Canada (2018 population ≈ 14.5 million) was associated with (1) decreased wait times for hip fracture surgery without the unintended consequences of; (2) increased wait times for other extremity fracture surgeries not funded through the QBP mechanism [16]; and/or (3) increased after-hours provision of hip fracture surgery[17]. We hypothesized that implementing targeted QBP funding for hip fracture surgery was associated with reductions in wait times for hip fracture surgery, increases in wait times for non-hip fracture surgery, and increases in the incidence of after-hours hip fracture surgery. We independently evaluated wait times for hip and non-hip fracture surgeries, and incidence of after hours hip fracture surgeries. Our hypothesis was informed by the government’s stated intent to optimize patient outcomes through QBPs, including by reducing wait times [12]. We reasoned that because QBP funding designated for hip fracture surgeries could only be used for that purpose, and because the clinical pathway was intended to help streamline care, hospitals would be incentivized to enable increased hip fracture surgery volumes including after-hours, thereby crowding out non-QBP-funded extremity fractures.

## Methods

### Study Design and Setting

We conducted an aggregate, population-based, interrupted time series (ITS) analysis of administrative health data from Ontario, Canada, where all medically necessary hospital and physician services are publicly-funded by a single provincial health insurance program.

### Data Sources

We used multiple population-wide administrative health databases linked with encoded identifiers to identify study patients and ascertain outcomes. Ontario’s ICES Data Repository consists of record-level, coded, linkable, health data sets, encompassing much of the publicly-funded administrative health services records for the population of Ontario. From this repository, we relied on aspects of the following data: patient demographic information, vital statistics, hospital discharge diagnosis, ICD-10 surgical procedures, and emergency department encounters. Patient demographic information and vital status were obtained from the Ontario Health Insurance Plan (OHIP) Registered Persons Database. The Canadian Institute for Health Information (CIHI) Discharge Abstract Database (CIHI-DAD) contains hospital discharge diagnoses that are coded using International Classification of Diseases 10th revision (ICD-10) and surgical procedures that are identified using Canadian Classification of Health Intervention (CCI) codes. The OHIP Claims History Database contains physician service claims with both diagnosis and procedure codes. Emergency department (ED) encounters were captured using the CIHI National Ambulatory Care Reporting System (NACRS). These datasets are held securely in a linked coded form and were analyzed at ICES in Toronto, Canada (www.ices.on.ca).

### Exposure

QBP funding for hip fracture surgery was implemented jurisdiction-wide on April 1, 2014, at the start of the fiscal year. On that date, the Ministry of Health switched funding for hip fracture surgeries from global budget to QBP.

### Identification of Patients

We identified all patients in Ontario, Canada undergoing surgery for hip fractures, ankle fractures, tibial plateau fractures, and appendectomy between April 1, 2012 and February 28, 2017. Codes used to identify patients in this study are available in the Table in the supplement. Surgeries for ankle and tibial plateau fractures served as control orthopaedic procedures, since they were not funded by QBPs, enabling us to assess effects on QBP- vs. non-QBP-funded surgeries. Appendectomy served as a non-orthopedic negative control because we did not expect this procedure to be influenced by the QBP funding reform, thereby allowing us to assess secular trends in the overall health care system. All three of these control procedures are funded under each hospital’s global budget, rather than from the targeted QBP funding envelope. Within each of these four cohorts, we excluded non-residents, those under 18 years of age, and patients with missing age, sex, or Ontario health card number. To avoid misclassification, we also excluded patients coded with elective procedures, patients waiting greater than 14 days for surgery, and repeat surgeries occurring within 30 days of hospital admission. Open fractures also were excluded as they are rare and unlikely to be influenced by funding reforms because they are managed urgently ahead of almost all other orthopaedic cases.

### Covariates

We reported age, sex, urban or rural location (defined by postal codes), and neighbourhood income quintile (derived from census data). Comorbidity was ascertained using the Deyo-Charlson Co-morbidity Index [21] and hospital encounter data over the 3 years preceding the index surgical admission. We also recorded each patient’s (1) health service utilization (emergency department encounters and hospitalizations) in the year prior to surgery, (2) prior place of residence (long-term care or not), and 3) treating hospital type (teaching versus community).

### Outcomes

We measured the mean (± standard deviation [SD]) from time of first presentation at hospital to time the surgical procedure began in hours. Percentage of surgeries performed within 24 h (a clinically relevant time threshold) was also assessed. Time of first presentation was defined as time of ED registration or hospital admittance (for admissions that bypassed an ED). Percentage of surgeries performed after-hours (defined as surgery beginning between 1700 and 0700 h) was also assessed. Timing of (1) first presentation at hospital and (2) start of surgery have been recorded in CIHI-DAD since 2009 (before our study period began) and have previously been used to calculate exact wait times for after-hours provision of surgery [10, 11, 13, 17, 18].

### Statistical Analysis

 We described patient characteristics stratified by surgical procedure and outcomes annually for each year in the study period. Segmented regression analysis was used to evaluate the statistical significance of changes in wait times and the percentage of after-hours cases for these procedures from before to after the funding policy change. The unit of time for the analysis was one month, with 24 monthly data points pre-policy and 36 monthly data points post-policy. Although QBP funding for all Ontario hospitals performing hip fracture surgeries began simultaneously on April 1, 2014, we excluded from analyses *a priori* the three months of data immediately following the start of the funding change (one fiscal quarter) to account for a policy “transition” period [19]. We reasoned that by censuring those first three months of observation after the start of the intervention, we could be confident that any observed changes from then on could be attributed to the funding policy change. An automatic fitting procedure in the R statistical software package was used to account for seasonality and determine the number of autocorrelation lags to include in the analysis [20]. We calculated the difference (and 95 % confidence intervals [CIs] for the difference) between the observed rate post-implementation and the predicted rate that would have occurred had QBPs been not implemented [21]. All analyses were performed at ICES using R version 3.4.4 and a two-sided type-I error probability of 0.05. Use of data in this study was authorized under Sec. 45 of Ontario’s Personal Health Information Protection Act, which does not require review by a Research Ethics Board.

## Results

Our study included 49,097 patients with hip fracture surgery, 10,474 with ankle fracture surgery, 1,594 with tibial plateau fracture surgery, and 40,898 with appendectomy (Supplementary Table). Table 1 presents the patient characteristics for each cohort at time of surgery. Patients with hip fractures had a mean (SD) age of 80.4 [12] years, which was older than patients within the control fracture and appendectomy cohorts. More females experienced hip and ankle fractures, whereas males and females were equally likely to undergo surgeries for tibial plateau fractures and appendectomy. Patients undergoing hip fracture surgery also had more comorbidities, ED visits, and days in hospital within the year leading up to their fracture. A higher percentage of patients with hip fractures resided in long term care residences (15 %) (compared to ≤1 % of patients in each of the 3 comparator cohorts). Most patients (> 80 % for all groups) were admitted to hospital from the ED. Within the fracture cohorts, the annual mean and median wait times from ED or hospitalization to surgery were shortest for surgeries for ankle fractures, followed by hip fractures, and longest for tibial plateau fractures (Table 2). Within the fracture cohorts, after hours surgery was commonly performed for hip fractures (in 36–39 % of cases) and ankle fractures (in 35–38 %) and only slightly less frequent for tibial plateau fractures (25–26 %).

**Table 1:** Characteristics of Study Patients

	hip fractures ***N***=49,097	ankle fractures ***N***=10,474	tibial plateau fractures ***N***=1,594	Appendectomy ***N***=40,898
**Age (mean ± SD)**	80.4 ± 12.1	53.9 ± 18.3	52.0 ± 16.0	40.4 ± 16.5
**Female**	34,122 (69.5%)	6,479 (61.9%)	855 (53.6%)	20,315 (49.7%)
**Neighbourhood income quintile**				
1 (lowest)	10,718 (21.8%)	2,130 (20.3%)	356 (22.3%)	7,614 (18.6%)
2	9,884 (20.1%)	2,050 (19.6%)	324 (20.3%)	7,780 (19.0%)
3	9,635 (19.6%)	2,019 (19.3%)	308 (19.3%)	7,962 (19.5%)
4	9,549 (19.4%)	2,205 (21.1%)	311 (19.5%)	8,846 (21.6%)
5 (highest)	9,041 (18.4%)	2,020 (19.3%)	287 (18.0%)	8,509 (20.8%)
**Living in a rural area**	6,559 (13.4%)	1,267 (12.1%)	282 (17.7%)	4,241 (10.4%)
**Charlson Index (mean ± SD)**	1.7 ± 1.8	1.1 ± 1.6	1.0 ± 1.6	0.5 ± 1.1
0	6,838 (13.9%)	1,091 (10.4%)	173 (10.9%)	4,717 (11.5%)
1	6,188 (12.6%)	365 (3.5%)	48 (3.0%)	616 (1.5%)
2	3,924 (8.0%)	253 (2.4%)	32 (2.0%)	437 (1.1%)
3	2,570 (5.2%)	171 (1.6%)	20 (1.3%)	131 (0.3%)
4	1,354 (2.8%)	69 (0.7%)	≤5	53 (0.1%)
≥5	1,812 (3.7%)	99 (0.9%)	17 (1.1%)	100 (0.2%)
**Number of ED visits**^**a**^**, median (IQR)**	2 (1-3)	1 (1-2)	1 (1-2)	1 (1-2)
**Number of hospitalization days**^**a**^**, median (IQR)**	10 (5-22)	5 (3-11)	4 (2-8)	3 (2-6)
**Method of entry into hospital:**				
From ED	43,108 (87.8%)	9,025 (86.2%)	1,327 (83.2%)	40,179 (98.2%)
Direct	5,989 (12.2%)	1,449 (13.8%)	267 (16.8%)	719 (1.8%)
**Long term care resident**	7,691 (15.7%)	117 (1.1%)	8 (0.5%)	42 (0.1%)
**Community hospital**	35,391 (72.1%)	7,276 (69.5%)	824 (51.7%)	30,440 (74.4%)
**Teaching hospital**	13,706 (27.9%)	3,198 (30.5%)	770 (48.3%)	10,458 (25.6%)

^a^in the past year; Abbreviations: *ED* Emergency Department; *IQR* interquartile range; *LTC* long term care; *SD* standard deviation

**Table 2:** Annual average wait times for surgery and number (%) of surgeries performed after hours

	April 2012 – March 2013	April 2013– March 2014	April 2014 – March 2015	April 2015 – March 2016	April 2016 – March 2017
**Mean ± SD Wait time in hours from ED/hospitalization to surgery**					
Hip fractures	37.6 ± 31.0	36.9 ± 30.7	35.8 ± 28.9	34.4 ± 27.2	35.4 ± 27.7
Ankle fractures	32.3 ± 33.8	32.0 ± 35.3	30.6 ± 32.9	31.5 ± 35.8	37.0 ± 41.4
Tibial plateau fractures	54.8 ± 58.9	53.5 ± 53.5	55.3 ± 60.0	63.7 ± 64.5	60.5 ± 56.3
Appendectomy	12.9 ± 10.1	13.0 ± 10.3	13.0 ± 10.6	13.4 ± 11.7	13.8 ± 12.5
**Operating time: After hours**^**a**^					
Hip fractures	3,680 (39.0%)	4,002 (39.0%)	3,674 (36.9%)	3,588 (36.0%)	3,448 (36.4%)
Ankle fractures	832 (36.4%)	896 (37.5%)	746 (36.7%)	668 (35.1%)	628 (33.7%)
Tibial plateau fractures	80 (25.3%)	79 (25.7%)	80 (25.8%)	86 (25.1%)	94 (29.5%)
Appendectomy	5,493 (68.8%)	5,473 (66.9%)	5,404 (66.1%)	5,630 (64.3%)	5,034 (64.4%)

^a^Between the hours of 17:00 to 7:00

**Fig. 1:**
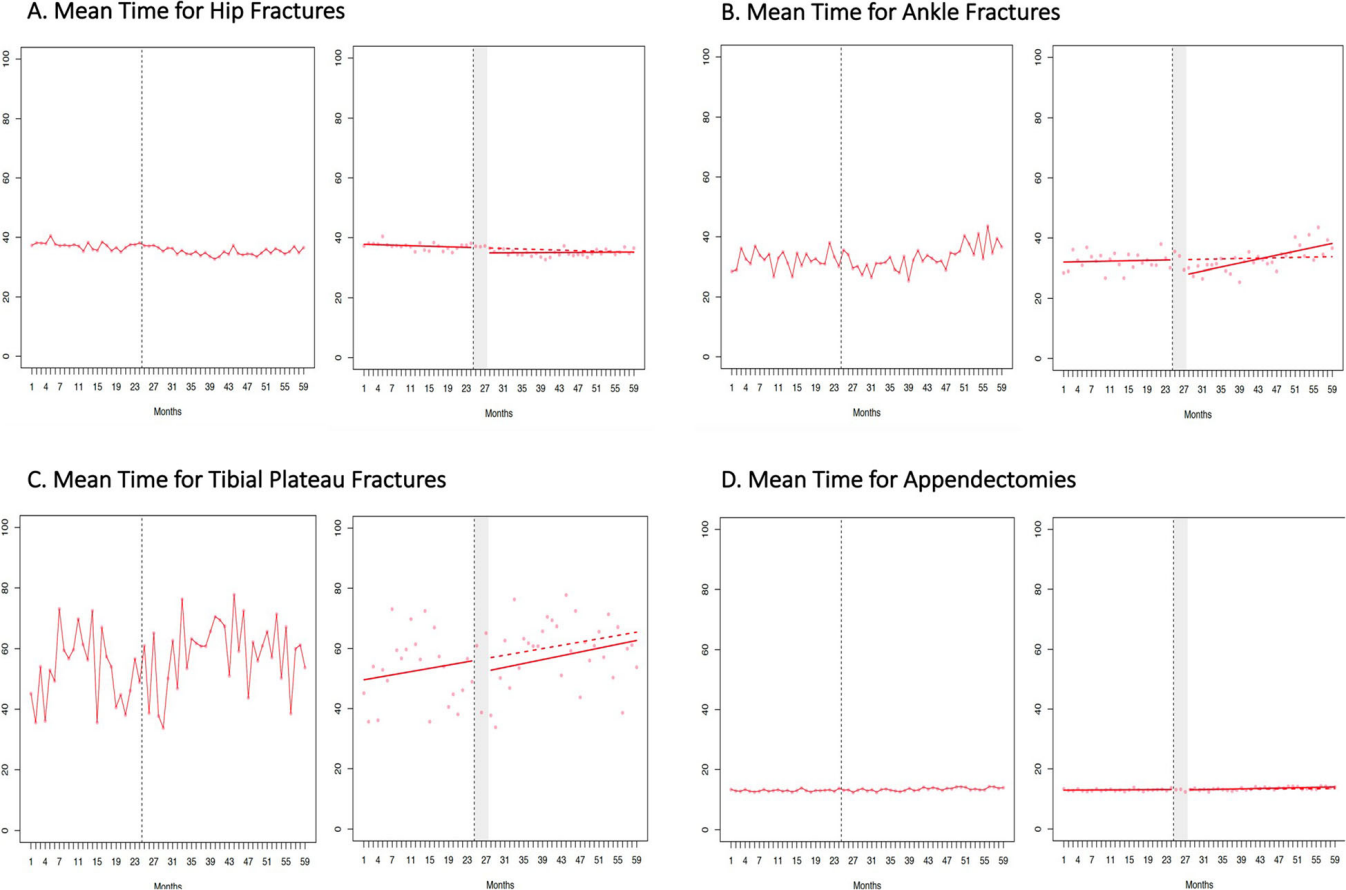
Monthly mean wait time (in hours) from hospital presentation to surgery over the study period. For A, B, C, and D, the interrupted time series analysis is displayed on the right. Red solid line represents the fitted model calculated by segmented regression. The red dashed line represents the counterfactual (i.e. if no policy change occurred). The grey vertical dashed line represents the date of policy change. The grey shaded area represents the 3-month “transition” period allowed for policy implementation. There was no statistically significant difference between the fitted model and counterfactual scenario for any surgery type (Table 3).

**Fig. 2:**
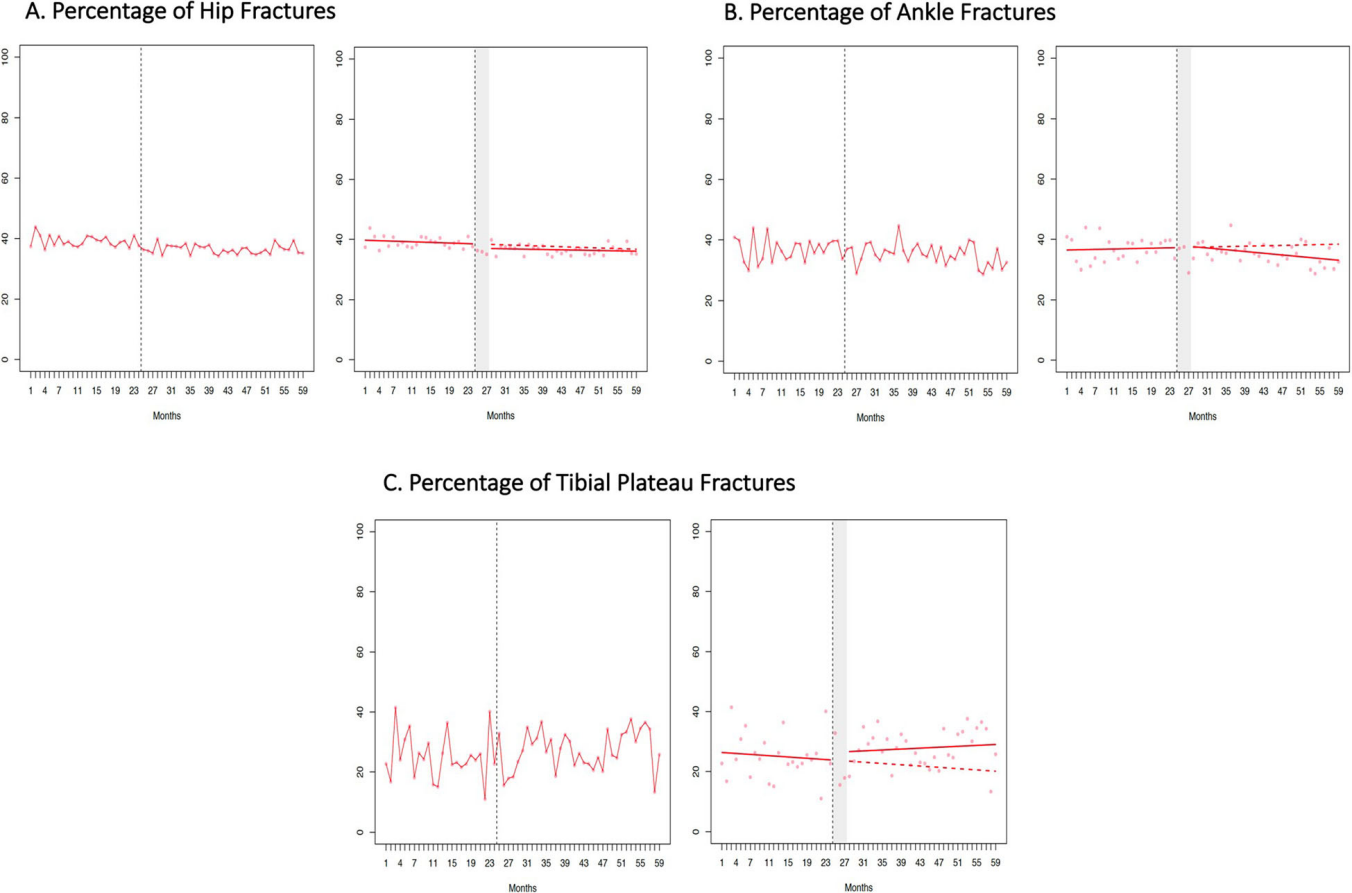
Percentage of surgeries (1700 – 0700 hours) performed after hours over the study period. For A, B, C, and D, the interrupted time series analysis is displayed on the right. Red solid line represents the fitted model calculated by segmented regression. The red dashed line represents the counterfactual (i.e. if no policy change occurred). The grey shaded area represents the 3-month “transition” period allowed for policy implementation. The grey shaded area represents the “transition” period. There was no statistically significant difference between the fitted model and counterfactual scenario for any surgery type (Table 3).

With the implementation of funding reform, there were no significant changes in wait times or provision of after-hours surgery for hip, ankle, or tibial plateau repair or appendectomy (Figs. 1 and 2; Table 3). The difference (95 % CI) between the actual mean wait time post-implementation and the predicted rate had QBPs not been implemented was − 0.46 h (-3.94 h, 3.03 h) for hip fractures, 1.46 h (-3.58 h, 6.50 h) for ankle fractures, -3.22 h (-39.39 h, 32.95 h) for tibial plateau fractures, and 0.33 h (-0.57 h, 1.24 h) for appendectomy (Fig. 1; Table 3). The difference (95 % CI) between the actual percentage of surgeries performed after-hours post-implementation and the predicted rate was − 0.90 % (-3.91 %, 2.11 %) for hip fractures, -3.54 % (-11.25 %, 4.16 %) for ankle fractures, 7.09 % (-7.97 %, 22.14 %) for tibial plateau fractures, and 1.07 % (-2.45 %, 4.59 %) for appendectomy (Fig. 2; Table 3). The percentage of hip fracture surgeries occurring within 24 h was also unchanged after implementation of funding reform [-0.13 % (-6.08 %, 5.82 %)].

**Table 3:** Estimated effect of implementation of QBPs on outcomes from the segmented regression analysis. Calculated as counterfactual difference from the difference (95% Confidence Intervals) between the actual rate post-implementation and the predicted rate had QBP not been implemented

	Estimate (95% CI)
**Mean wait time (in hours) from ED/hospital admission to surgery**	
Hip fractures	- 0.46 (-3.94, 3.03)
Ankle fractures	1.46 (-3.58, 6.50)
Tibial plateau fractures	-3.22 (-39.39, 32.95)
Appendectomy	0.33 (-0.57, 1.24)
**Percentage (%) of surgeries performed after hours**^**a**^	
Hip fractures	-0.90 (-3.91, 2.11)
Ankle fractures	-3.54 (-11.25, 4.16)
Tibial plateau fractures	7.09 (-7.97, 22.14)
Appendectomy	1.07 (-2.45, 4.59)

^a^After hours = evening or overnight (between the hours of 1700 – 0700, any day)

## Discussion

 We evaluated the effects of a new hospital funding model on surgical care in this population-based cohort study in Ontario, Canada. We found no significant effects of this policy change on mean hip fracture surgery wait times. The policy change also did not significantly increase wait times for selected non-hip fracture surgeries or the frequency of after-hours surgeries performed. Our findings have important implications for health care system reform as policymakers worldwide experiment with new funding models.

First, other approaches for improving health system performance may be worth pursuing alongside, or instead of, funding reform. Like all “patient focused” variants of activity-based funding, QBPs established a prospective payment rate based on service type and volume. Funding was carved out of hospitals’ global budgets and then reallocated to hospitals at the start of the relevant fiscal year as a fixed price and fixed volume, targeted at each QBP procedure or diagnosis. QBPs may have failed to reduce wait times for hip fracture surgery or standardize care because: (i) QBPs specifically, and funding reform generally, were not the right “tool for the job”; or (ii) the way in which they were implemented compromised their success; or (iii) there were other unexamined contextual factors that may have explained the lack of effect and which may have influenced the circumstances in which QBPs might have produced the desired effects. QBPs differ from most typical ABF reforms in that funding applies only to a very limited set of diagnoses and procedures, and they rely on the voluntary use of handbooks to encourage incorporation of best practices. There is no mechanism in place to enforce (or systematically measure) adherence to the clinical pathways; hospitals are paid via QBPs whether they follow the pathways or not, but the intent was that following the pathways would enable hospitals to deliver care for the amount paid by QBPs. Indeed, prior qualitative work has indicated that QBPs coupled with dissemination of clinical handbooks were not effective at establishing consistent standards of practice [2].

Funding reform also may not be the best way to incentivize changes needed to decrease length of stay and increase patient throughput, especially if existing pressures to make these changes are already strong and/or if the performance levels achieved in this area are already near-optimal given existing constraints [22]. On the one hand, this apparent lack of effect is disappointing given that an excess of 66 % of hip fracture patients in Ontario do not receive surgery within the safe time frame of 24 h [11]. On the other hand, Ontario already out-performs the Canadian averages at present: in a 2018 nationwide study, 77 % of Canadians do not undergo hip fracture surgery within 24 h [14]. Previous qualitative analyses identified delays and others challenges in implementing QBPs in hospitals, which altered the trajectory of this funding reform [2]. Within orthopedic surgery, health care leaders’ early implementation experiences with the QBP for hip and knee replacements, which predated the QBP for hip fractures, identified lack of organisational preparedness as a major barrier [23].

This study has several strengths. Interrupted time series analyses provide a robust quasi-experimental design for inferring causality between health policy interventions and outcomes in the absence of a randomized controlled trial [24]. ITS was ideal given that a difference-in-difference approach was not appropriate since matched controls were not available from the Ontario data. Additional strengths of our study include the single-payer nature of Canada’s publicly-funded health care system, which captures all acute non-elective hip fracture surgeries performed across publicly-funded hospitals in Ontario, and the ability to assess wait times and timing of procedures through standardized data available from a single repository at ICES. The use of control procedures further strengthens our study and suggest a lack of other health system changes targeted at wait times concurrent with the implementation of QBPs. The study was powered to pick up differences, should they have existed, with 24 monthly data points pre-policy and 36 monthly data points post-policy. Excluding open fractures, elective procedures and very long delays avoided misclassification that would have also biased towards finding no differences with policy change.

Some limitations merit emphasis. First, our analyses were confined to health administrative data which lack clinical details to accurately identify more complex patients who are predisposed to both complications and longer wait times to optimize medical status prior to surgery. Second, our study did not assess the effects of other earlier health system interventions impacting wait times, such as the public reporting of wait time performance benchmarks which began in 2009, or the earlier dissemination of hip fracture surgery recommendations in 2005 [25]. However, unless coding practices, clinical care, or patient characteristics changed suddenly close to the time of QBP implementation, which we have no evidence of, any background/secular trends are accounted for in our analysis and pose no threat to the validity of study. Third, patient reported outcome and experience measures (PROMs and PREMs) were not available to us, and it is unknown whether the best practice clinical pathways disseminated for the hip fracture QBP [12] led to improvements in such outcomes. Future research to inform reform efforts could include evaluation of PROMS and PREMS, analysis of the extent to which clinicians voluntarily comply with clinical pathways and/or digital QBP clinical order sets, and investigation of the degree to which funding reform aligns with the intrinsic factors that motivate behaviour in clinicians and health care leaders [26].

## Conclusions

The implementation of a novel funding model for hospitals, based on fixed price and volume for targeted procedures and diagnoses, resulted in no significant system-wide effects on hip fracture surgery wait times. The policy change also did not appear to have negative consequences on wait times for other fracture surgeries or in frequency of after-hours surgery. Our findings suggest that other approaches for improving health system performance may be worth pursuing alongside, or instead of, funding reform.

## Supplementary Information

**Additional file 1:Supplementary Table** Description of the Derivation of Each Cohort

## Data Availability

We used population-wide administrative health databases linked at Ontario’s Institute for Clinical Evaluative Sciences (ICES; https://www.ices.on.ca/). Public access to these databases are closed and use of data in this study was authorized under Sec. 45 of Ontario’s Personal Health Information Protection Act.

## Abbreviations

QBPs: Quality-Based Procedures
ABF: Activity-based funding
ITS: Interrupted time series
PROMs and PREMs: Patient reported outcome and experience measures
