# Supplementary material for: Effects of hospital funding reform on wait times for hip fracture surgery: a population-based interrupted time-series analysis
Source: BMC Health Serv Res. 2021 Jun 13;21:576. doi: 10.1186/s12913-021-06601-2 (PMC8201723; doi:10.1186/s12913-021-06601-2)
Supplement: Supplementary file 1 — Additional file 1:Supplementary Table Description of the Derivation of Each Cohort [file 12913_2021_6601_MOESM1_ESM.docx]

**Supplementary Table. Description of the Derivation of Each Cohort**

|  | **Index Procedure** |  | **Control Procedures** | |  | **Negative Tracer** |
| --- | --- | --- | --- | --- | --- | --- |
|  | hip fractures |  | ankle fractures | tibial plateau fractures |  | appendectomy |
| Total Number of Procedures^1^ Performed between 2012-2017 | N=146,109 |  | N=16,455 | N=3,326 |  | N=67,470 |
| **Reasons for Exclusion:** |  |  |  |  |  |  |
| Missing main diagnosis code^2^ | 90,576(62.0%) |  | N/A | N/A |  | 7,211 (10.7%) |
| Invalid HCN, date of birth, or sex | 1,503 (1.0%) |  | 452 (2.7%) | 74 (2.2%) |  | 2,764 (4.1%) |
| Missing OHIP feecode^3^ | 2,374 (1.6%) |  | 1,973 (12.0%) | 1,015 (30.5%) |  | 3,181 (4.7%) |
| Non-Ontario resident | 61 (0.0%) |  | 12 (0.1%) | ≤5 |  | 63 (0.1%) |
| Age < 18 or age > 105 | 108 (0.1%) |  | 614 (3.7%) | 54 (1.6%) |  | 12,649 (18.7%) |
| NOT HIG^4^ | 255 (0.2%) |  | N/A | N/A |  | N/A |
| Episode separated by 30 days | 124 (0.1%) |  | 52 (0.3%) | 9 (0.3%) |  | N/A |
| Elective surgery | 1,489 (1.0%) |  | 1,465 (8.9%) | 359 (10.8%) |  | 572 (0.8%) |
| Non-ED or direct hospital admission | 110 (0.1%) |  | 519 (3.2%) | 56 (1.7%) |  | 116 (0.2%) |
| Procedure ≥ 2 weeks after ED | 262 (0.2%) |  | 77 (0.5%) | 41 (1.2%) |  | 6 (0.0%) |
| Open fracture | 65 (0.0%) |  | 816 (5.0%) | 123 (3.7%) |  | N/A |
| Total Excluded: | 97,012 (66.4%) |  | 5,981 (36.3%) | 1732 (52.1%) |  | 26,572 (39.4%) |
| **Final Numbers of Acute Non-Elective Procedures** | **N=49,097** |  | **N=10,474** | **N=1,594** |  | **N=40,898** |
| ^1^According to Canadian Classification of Health Interventions (CCI) procedure codes in Hospital Discharge Abstract Data for Hip Fracture (1VA53 1VA74 1VA80 1VC74 1VC80), ankle fractures (1WA74),tibial plateau fractures (1VG74), appendectomy (1NV89); ^2^ICD 10 diagnosis codes as the main diagnosis for hip fracture (S72.0, S72.1, S72.2, excluding S72.00 unspecified fracture) and Acute Appendicitis (K35). Please note 90,576(62.0%) records were excluded because they represented hip replacements performed for conditions other than hip fracture (including osteoarthritis); ^3^ Missing OHIP procedure fee code +/- 7 days to verify service provider; ^4^Not Health Based Allocation Model (HBAM) Inpatient Group (HIG): ‘726’, ‘727’, ‘766’;  Abbreviations: ED = Emergency Department; HCN = health card number; N/A = not applicable (criteria was not applied); | | | | | | |
